# One-Pot Visual Detection of African Swine Fever Virus Using CRISPR-Cas12a

**DOI:** 10.3389/fvets.2022.962438

**Published:** 2022-07-18

**Authors:** Chao Qin, Jiajia Liu, Wenqi Zhu, Muchu Zeng, Ke Xu, Jinmei Ding, Hao Zhou, Jianshen Zhu, Yuqing Ke, Lai Yan Li, Gaoyuan Sheng, Zhuoru Li, Huaixi Luo, Shengyao Jiang, Kangchun Chen, Xianting Ding, He Meng

**Affiliations:** ^1^Shanghai Key Laboratory of Veterinary Biotechnology, Department of Animal Science, School of Agriculture and Biology, Shanghai Jiaotong University, Shanghai, China; ^2^State Key Laboratory of Oncogenes and Related Genes, School of Biomedical Engineering, Institute for Personalized Medicine, Shanghai Jiaotong University, Shanghai, China

**Keywords:** CRISPR/Cas12a, detection, African swine fever virus, RPA, LAMP

## Abstract

African swine fever virus (ASFV) is a leading cause of worldwide agricultural loss. ASFV is a highly contagious and lethal disease for both domestic and wild pigs, which has brought enormous economic losses to a number of countries. Conventional methods, such as general polymerase chain reaction and isothermal amplification, are time-consuming, instrument-dependent, and unsatisfactorily accurate. Therefore, rapid, sensitive, and field-deployable detection of ASFV is important for disease surveillance and control. Herein, we created a one-pot visual detection system for ASFV with CRISPR/Cas12a technology combined with LAMP or RPA. A mineral oil sealing strategy was adopted to mitigate sample cross-contamination between parallel vials during high-throughput testing. Furthermore, the blue fluorescence signal produced by ssDNA reporter could be observed by the naked eye without any dedicated instrument. For CRISPR-RPA system, detection could be completed within 40 min with advantageous sensitivity. While CRISPR-LAMP system could complete it within 60 min with a high sensitivity of 5.8 × 10^2^ copies/μl. Furthermore, we verified such detection platforms display no cross-reactivity with other porcine DNA or RNA viruses. Both CRISPR-RPA and CRISPR-LAMP systems permit highly rapid, sensitive, specific, and low-cost Cas12a-mediated visual diagnostic of ASFV for point-of-care testing (POCT) applications.

## Introduction

African swine fever (ASF) is a highly lethal and contagious disease in domestic pigs caused by the African swine fever virus (ASFV) and is a notifiable disease by the World Organization for Animal Health (OIE) (1). ASFV is a large and complex double-stranded DNA arbovirus that is the only member of *Asfivirus* genus (2). Based on the highly conserved gene *B646L* encoding the viral protein p72, ASFV is currently classified into 24 genotypes. In 2018, ASF began to spread in China, where the virus circulating was identified as genotype II (1, 3), an epidemic strain that was 100% consistent with that in Russia (4). Due to the absence of effective treatments or vaccines, ASF disease control mainly relies on culling pigs (2, 5). With high infectivity and mortality, ASFV has seriously affected animal husbandry.

The focus of prevention and control of ASF is currently still in the early diagnosis and outbreak control stages. Therefore, accurate and efficient laboratory diagnosis is of vital importance. Quantitative PCR (qPCR) and conventional PCR, which are also recommended by OIE (6–8), are sensitive methods for the detection of ASFV. However, the dependence on expensive thermocyclers and skilled operators limits the application of these methods for point-of-care (POC) detection (9, 10). Isothermal amplification techniques, such as recombinase polymerase amplification (RPA) (11–13), loop-mediated isothermal amplification (LAMP) (14, 15), polymerase cross-linking spiral reaction (PCLSR) (16), and cross-priming amplification (CPA) (17), have been suggested for the detection of ASFV based on the highly conserved region of essential ASFV genes, such as *p72*. However, the reaction temperature close to room temperature can easily generate false-positive test results (18, 19). Therefore, it is necessary to develop a sensitive, specific, equipment-free, and visual method for the detection of ASFV.

Recently, clustered regularly interspaced short palindromic repeat (CRISPR)-associated endonuclease (CRISPR/Cas) systems have been developed to detect nucleic acid, including Cas13a (20–26), Cas12a (27–31), Cas9 (32–34), Cas12b (35), and Cas14 (36). Cas12a, an RNA-guided DNA endonuclease, recognizes a T nucleotide rich, such as 5′- (T) TTN-3′, protospacer-adjacent motif (PAM) with the help of CRISPR RNA (crRNA) (37). Then using a single RuvC catalytic domain (38–40), the Cas12a generates a 5′-overhang staggered cut on the target strand (TS) and the non-target strand (NTS). Both the crRNA-complementary ssDNA or dsDNA (the activator) activate the trans-cleavage to unleash the robust, non-specific ssDNA trans-cleavage activity of Cas12a. The Cas12a-based detection can be performed at physiological temperature or even at room temperature; meanwhile, the non-specific cleavage by these Cas12a enzymes occurs at a very high turnover rate (41–43), significantly simplifying the detection procedure and enabling the accurate detection of low-concentration targets. Combined with fluorophore quencher (FQ)–labeled reporter, the diagnostic technology based on CRISPR/cas12a has been successfully applied for the detection of the porcine reproductive and respiratory syndrome virus (PRRSV) (44), white spot syndrome virus (WSSV) (45), tobacco curly shoot virus (TCSV) (46), human papillomavirus 16 (HPV-16), parvovirus B19 (PB-19) (47), *Listeria monocytogenes* (48), foodborne bacteria (*Escherichia coli* and *Streptococcus aureus*) (49), and *Mycobacterium tuberculosis* (50).

To improve the convenience of existing tools and overcome the limitations of ASF diagnosis, a one-pot visual detection that integrates the CRISPR/Cas12a system with isothermal amplification has been developed in this study. The one-pot detection is sensitive, specific, low-cost, user-friendly, and ready to be used for on-site ASFV detection or other DNA-based pathogens.

## Materials and Methods

### Preparation of Genomic DNA Samples

The 1941bp fragment (Genomic Sequence: NC_001659.2) of the ASFV *p72* gene (also known as *B646L*) was chemically synthesized and cloned into pUC57 plasmid (herein referred to as pUC57-p72 DNA) by Sangon Biotech (Shanghai). Co. Ltd. The pUC57-p72 DNA was used as the template for the optimization of the detection system, as well as for the determination of sensitivity, as standard ASFV plasmid was used in previous reports (16, 17). The DNA or cDNA of the pseudorabies virus (PRV), porcine reproductive and respiratory syndrome virus (PRRSV), porcine epidemic diarrhea virus (PEDV), and porcine deltacoronavirus (PDCoV) were obtained from the Shanghai Veterinary Research Institute (Chinese Academy of Agricultural Sciences), for the use as samples for specificity determination.

### Oligonucleotide Primers for Amplification and crRNA Preparation

The most conserved region of the gene was subjected to design isothermal amplification primers. The RPA primers were designed using online software (Primer-blast) according to the TwistAmp assay. These forward and reverse primers formed a number of primer pairs, and the RPA products should be 100–200 bp. The LAMP primers were designed using PrimerExplorer V5 (http://primerexplorer.jp/e/), comprising of two outer primers F3 and B3, two inner primers FIP and BIP, and two loop primers LF and LB.

Using CHOPCHOP (https://chopchop.cbu.uib.no/), the 23nt crRNA targets were designed targeting the *p72* gene, which were also the targets of the RPA and LAMP. The primers and crRNAs sequences, listed in Table 1, were synthesized by Sangon Biotech.

**Table 1:** Sequence of primers, crRNA, and FQ reporter in this study.

**Name**	**Sequence (5^′^-3^′^)**
RPA-F	CGCAAATTTTGCATCCCAGGGGATAAAATGACTG
RPA-R	GGATATTGTGAGAGTTCTCGGGAAAATGTTGTGA
LAMP-F3	CGCAAATTTTGCATCCCA
LAMP-B3	GGATATTGTGAGAGTTCTCGG
LAMP-FIP	GAGAGGGCCACTAGTTCCCTAAAATGACTGGATATAAGCACTT
LAMP-BIP	CAAGCCGCACCAAAGCAAACGAATTTCGGGTTGGTATGG
LAMP-LF	ACCGATACCTCCTGGCCGAC
LAMP-LB	TCTTACCGATGAAAATGATACGCAG
crRNA	UAAUUUCUACUAAGUGUAGAUCACAAGCCGCACCAAAGCAAACC
ssDNA reporter	6-FAM/TTATT/BHQ-1

### RPA, LAMP Assays, and Cas12a/crRNA Nucleic Acid Detection

The RPA reaction was conducted using an RPA reaction mixture (TwistDx) containing 1.2 μl of primer RPA-F (10 μM), 1.2 μl of primer RPA-R (10 μM), 15 μl of primer-free rehydration buffer, 5.35 μl of ultrapure water, 1.25 μl of magnesium acetate (280 mM, MgOAc), and 1 μl of the template DNA or cDNA. The RPA reaction was incubated by PTC-200 thermocyclers (BIO-RAD) at a constant temperature of 37°C for 20 min.

The LAMP assay with the above designed LAMP primers was performed in a 25 μL reaction mixture containing 2.5 μl of 10× reaction buffer [200 mM Tris-HCl, 100 mM (NH_4_)_2_SO_4_, 500 mM KCl, 80 mM MgSO_4_, and 1% Tween 20], 2.5 μl of 10× LAMP primer mix [1.6 μM each of forward inner primer (FIP) and backward inner primer (BIP), 0.2 μM each of forward outer primer (F3) and backward outer primer (B3), 0.4 μM each of forward loop primer (FP) and backward loop primer (BP)], 3 μl of each dNTP (10mM), 4 μl of Betaine (5 M), 1 μl of Bst3.0 DNA polymerase (New England Biolabs), and 1 μl of the DNA or cDNA. The LAMP reaction was incubated by PTC-200 thermocyclers at a constant temperature of 65°C for 60 min.

The CRISPR/Cas12a-mediated cleavage assay (CRISPR reaction buffer) contained 2.5 μl of LbaCas12a (1 μM), 2.5 μl of crRNA (1 μM), 3 μl of ssDNA-FQ reporter (10 μM), 2 μl of RNase inhibitor (4U/μl), 4 μl of 10× NEBuffer, and 6 μl of ultrapure water. LbaCas12a, RNase inhibitor, and 10× NEBuffer were purchased from New England Biolabs, while the ssDNA-FQ reporter was synthesized with several nucleotides (5′-TTATT-3′) labeled with FAM at the 5′ end and a quencher at the 3′ end. The reaction was incubated in PTC-200 thermocyclers for 40 min at 37 °C. In addition, the fluorescence signal of the ssDNA–FQ report was visualized by the 2500B transilluminator under blue light (Tanon).

### Establishment and Optimization of the One-Pot Visual Detection System

**Figure 1:**
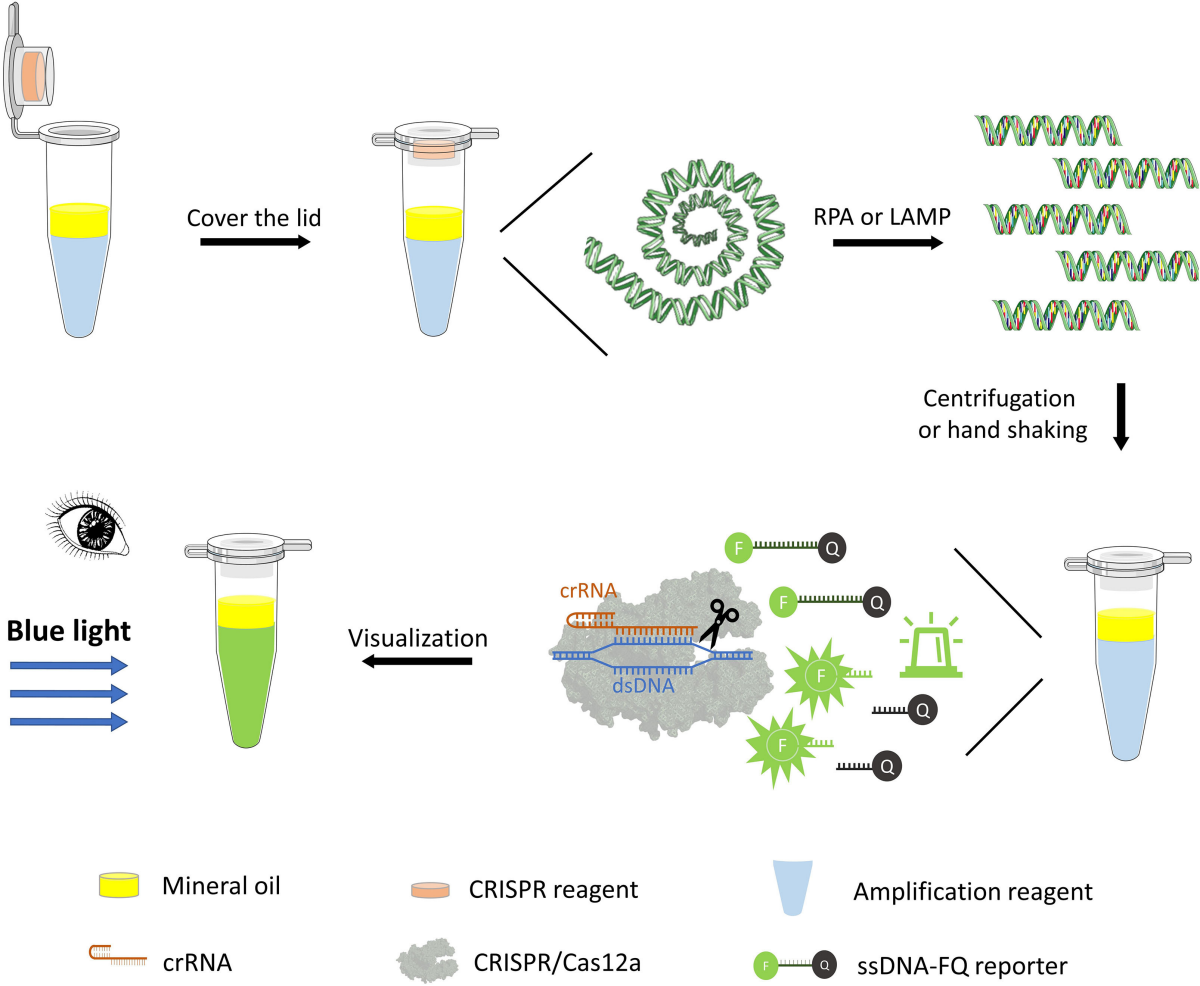
One-pot visual detection system. One-pot visual detection system showing the process of DNA pre-amplification, Cas12a/crRNA cleavage reaction, and fluorescence visualization. RPA or LAMP reagent, crRNA, Cas12a enzyme, and ssDNA-FQ reporter are all in one tube, with mineral oil sealing. Target binding of Cas12a will unleash its ability to digest ssDNA-FQ reporter. The fluorophore FAM **(F)** is quenched by a quencher **(Q)** if intact and emits fluorescence when cleaved, which is visual under the blue light.

One-pot detection combines isothermal pre-amplification and CRISPR/Cas12a-mediated cleavage detection in the same reaction tube. Briefly, the RPA or LAMP pre-amplification assays were added to an Eppendorf tube, and 35 μl of mineral oil was covered on the pre-amplification assay. After the RPA or LAMP reaction, 20 μl of CRISPR reaction buffer (pre-added inside the lid) was mixed with 25 μl of the amplification assay by hand shaking or spinning down in a minifuge for 5 s. The tube was put in PTC-200 thermocyclers at 37°C for 20 min, and the fluorescence signal after the CRISPR/Cas12a-mediated cleavage reaction was visualized using a transilluminator under blue light (Figure 1).

For the Cas12a-RPA one-pot detection system, a CRISPR reaction optimization experiment (incubated at 37°C for 5, 10, 20, and 30 min) was conducted to find an appropriate reaction time to shorten the one-pot detection. In addition, with a transfer step in between, the RPA and CRISPR reactions can be run sequentially. To optimize the one-pot detection, a single combined mixture of RPA and CRISPR reactions was carried out at the same reaction temperature (37 °C), which was “one-step” in one-pot detection. After 35 μl of mineral oil was used to cover the surface of the combined mixture, the reaction tube was put in PTC-200 thermocyclers at 37 °C for 20, 30, 40, and 50 min to optimize the detection system.

The reaction time of the CRISPR-LAMP one-pot detection system was optimized separately for different temperatures (65°C for LAMP and 37°C for CRISPR reaction). Briefly, LAMP pre-amplification was incubated at 65°C for 20, 30, 40, and 50 min, and CRISPR/Cas12a-mediated cleavage reaction was incubated at 37°C for 5, 10, 15, 20, 25, and 30 min after an optimized LAMP reaction time in one tube.

### Evaluation of the One-Pot Visual Detection System

The specificity of the one-pot visual detection systems was determined by the optimized procedure, the genomic DNA or cDNA of PRV, PRRSV, PEDV, and PDCoV. Further, pUC57-p72 DNA and negative controls (ddH_2_O) were also used, with the amount of genomic DNA or cDNA being 1 μl per reaction. In addition, all the genomic DNA or cDNA were used as templates for the PCR with their own specific primers (Supplementary Table 1) before evaluating the specificity of the one-pot visual detection system.

The analytic sensitivities of the newly developed one-pot visual detection systems were determined with the pUC57-p72 DNA ranging from 7 × 10^9^ to 7 × 10^0^ copies/μl using multiple dilution methods. The reaction mixtures were heated at 37 °C or 65 °C for an optimal amount of time. The ASFV *B646L* gene plasmid reference material [GBW(E) 091034], with high stability and uniformity, as well as an extended uncertainty of 0.9 × 10^3^ copies/μl (K = 2), was used for further determination of the sensitivity of both the CRISPR-RPA and CRISPR-LAMP one-pot detection systems. The reference material was diluted, ranging from 5.8 × 104 to 5.8 × 10^0^ copies/μl, and detected as previously described in the method section.

For further evaluation, diluted pUC57-p72 DNA was used as a template to compare the newly developed one-pot visual detection system with SYBR real-time qPCR. Briefly, 10 μl of 2× ChamQ Universal SYBR qPCR Master Mix (Vazyme, Nanjing), 0.4 μl of primer F (10 μM), 0.4 μl of primer R (10 μM), 2 μl of DNA, and ddH_2_O were included. The reactions were conducted in a 20 μl volume following the kit instructions. The reaction cycle parameters were set as denaturation at 95°C for 30 s, followed by 40 cycles of amplification, 95°C for 10 s and 60°C for 30 s in a CFX Connect fluorescence quantitative PCR detection system (BIO-RAD). The primers are listed in Supplementary Table 1, and the result was analyzed by GraphPad Prism 8.3.0.

## Results

### Establishing the One-Pot Detection Assay

**Figure 2:**
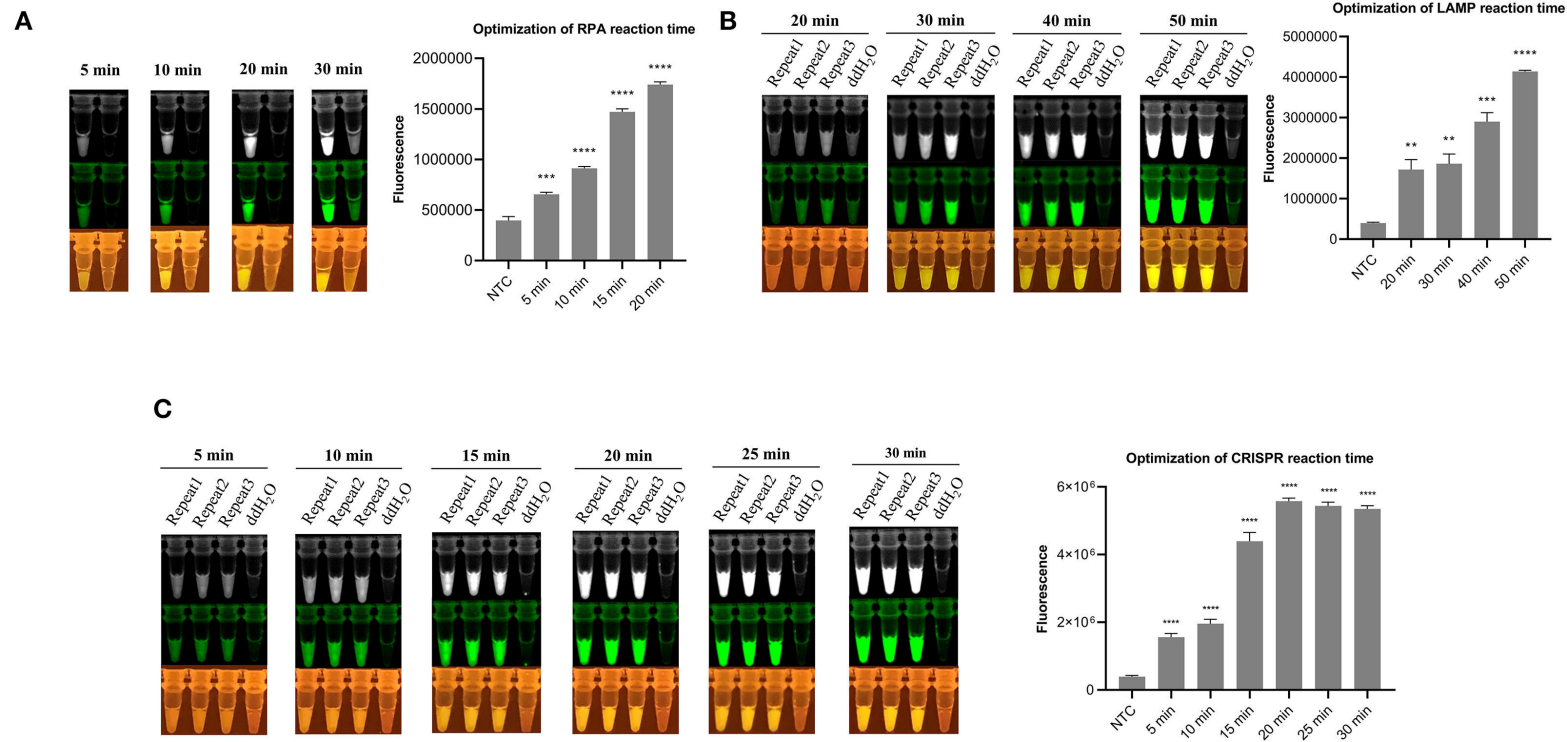
Fluorescence signal image in optimization of detection system. **(A)** Optimization of RPA reaction time in CRISPR-RPA one-pot detection (5, 10, 15, and 20 min). **(B)** Optimization of LAMP reaction time in CRISPR-LAMP one-pot detection (20, 30, 40, and 50 min). **(C)** Optimization of CRISPR reaction time in CRISPR-LAMP one-pot detection (5, 10, 15, 20, 25, and 30 min). The fluorescence signal monochrome and pseudo green images were taken from Tanon 2000M camera, and the images at the bottom were taken behind the UV/blue light resistant observation window. Analysis of the value for the fluorescence image using the ImageJ software. Each measuring was run with three replicates (*n* = 3). *P < 0.05, **P < 0.01, ***P < 0.001, ****P < 0.0001, ns, not significant. Error bars represent the means ± s.d. from replicates. The unpaired two-tailed *t*-test was used to analyze the statistical significance.

By adding isothermal pre-amplification and Cas12a-mediated cleavage reaction together in one tube, with mineral oil covering the surface of the LAMP/RPA amplification reagent, a one-tube visual detection system was assembled. For optimizing the CRISPR-RPA one-pot detection, the result (Figure 2A) showed that the fluorescence signal increased rapidly with time until it reached the peak value while the negative control remained no fluorescence signal. Moreover, the fluorescence signal was clear enough for the naked eye to detect at 20 min. Therefore, a total of 40 min (RPA for 20 min and CRISPR reaction for 20 min) reaction time were established for the CRISPR-RPA one-pot detection. Besides, after 40 min reaction time, the “one-step” in one-pot detection also showed an increased pattern in the same fluorescence signal until reaching the peak value (Supplementary Figure 1).

For the CRISPR-LAMP one-pot detection system, the optimization was separated into two parts, LAMP optimization and CRISPR reaction optimization. After incubating at 65°C for different durations and a Cas12a-mediated reaction for 40 min, the result (Figure 2B) showed that 40 min is an appropriate pre-amplification time for LAMP. After LAMP pre-amplifying for the optimized time (40 min), a CRISPR reaction was conducted to adjust the time. The result (Figure 2C) showed that 20 min is an optimized time for CRISPR reaction. Eventually, a total of 60 min (40 min for LAMP and 20 min for CRISPR reaction) reaction time were established for CRISPR-LAMP one-pot detection.

### Specificity of the One-Pot Visual Detection System

**Figure 3:**
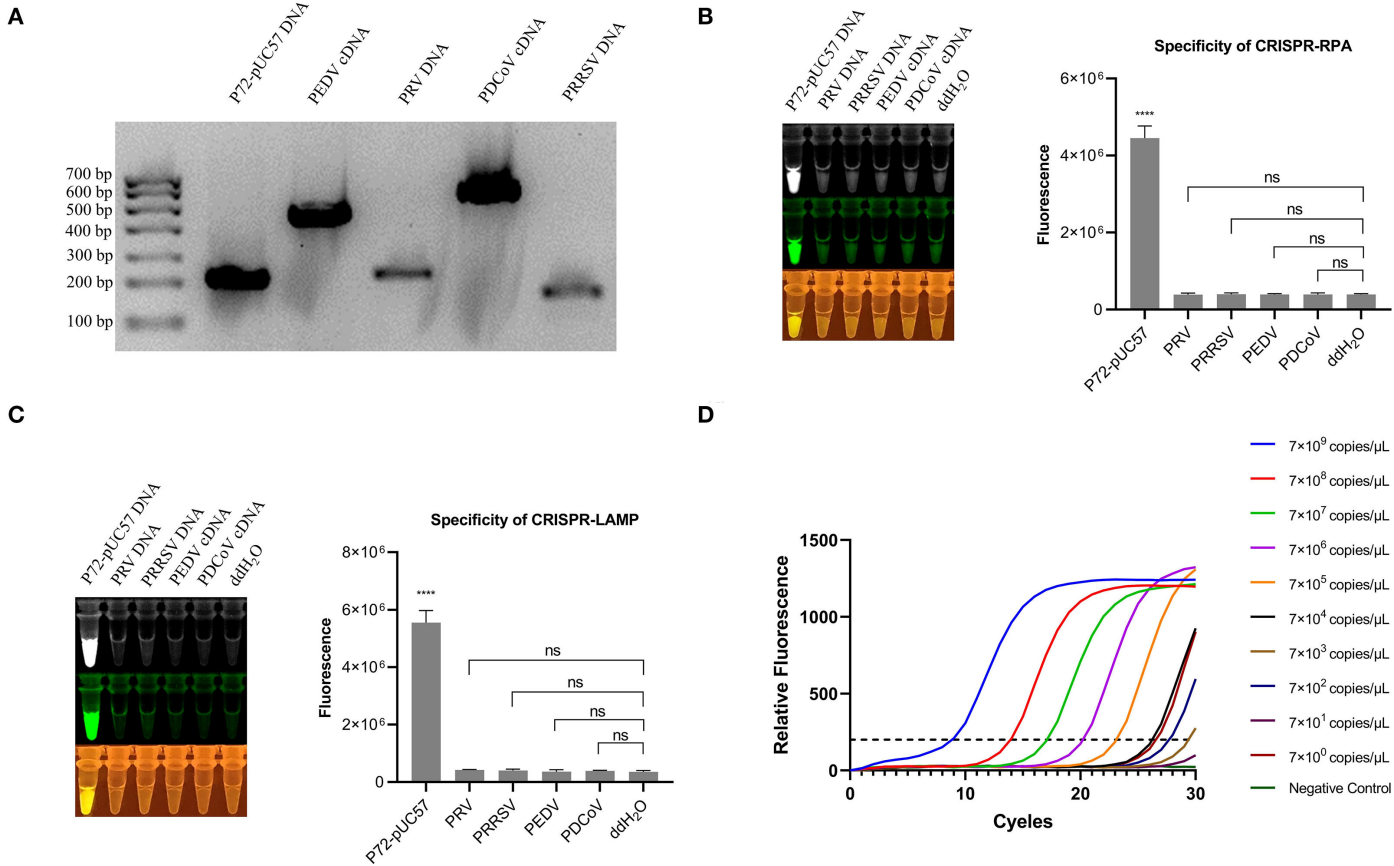
Specificity of one-pot visual detection system and qPCR. **(A)** PCR amplification result of pUC57-p72 and porcine virus genomic DNA or cDNA. **(B)** Specificity of CRISPR-RPA one-pot detection with pUC57-p72 and porcine virus genomic DNA or cDNA. **(C)** Specificity of CRISPR-LAMP one-pot detection with pUC57-p72 and porcine virus genomic DNA or cDNA. **(D)** qPCR for 10-fold serial diluted pUC57-p72 DNA (qPCR was processed using GraphPad 8.0). Analysis of the value for the fluorescence image used the ImageJ software. Each measuring was run with three replicates (*n* = 3). *P < 0.05, **P < 0.01, ***P < 0.001, ****P < 0.0001, ns, not significant. Error bars represent the means ± s.d. from replicates. The unpaired two-tailed *t*-test was used to analyze the statistical significance.

The specificity of the one-pot visual detection system was evaluated using pUC57-p72 DNA and other porcine viruses' genomic DNA or cDNA, including PRV, PRRSV, PEDV, and PDCoV. The specific of all the genomic DNA or cDNA were verified with PCR and the results of which are displayed in Figure 3A. For both the CRISPR-RPA and CRISPR-LAMP one-pot visual detections, after the template DNA was added and the tubes were incubated at a suitable temperature in the reaction order, the rapid reaction of pUC57-p72 DNA occurred and a strong fluorescence signal appeared while other genomic DNA or cDNA showed no signal (Figures 3B,C), which revealed there was no cross-reaction with other viruses. The results demonstrated that both CRISPR-RPA and CRISPR-LAMP one-pot visual detection systems could be used for specific detection of ASFV.

While estimating the sensitivity and specificity of the one-pot visual detection system, it showed high specificity and accuracy, as well as low detection limit, particularly the limit of CRISPR-LAMP was much lower.

### Sensitivity of the One-Pot Visual Detection System

**Figure 4:**
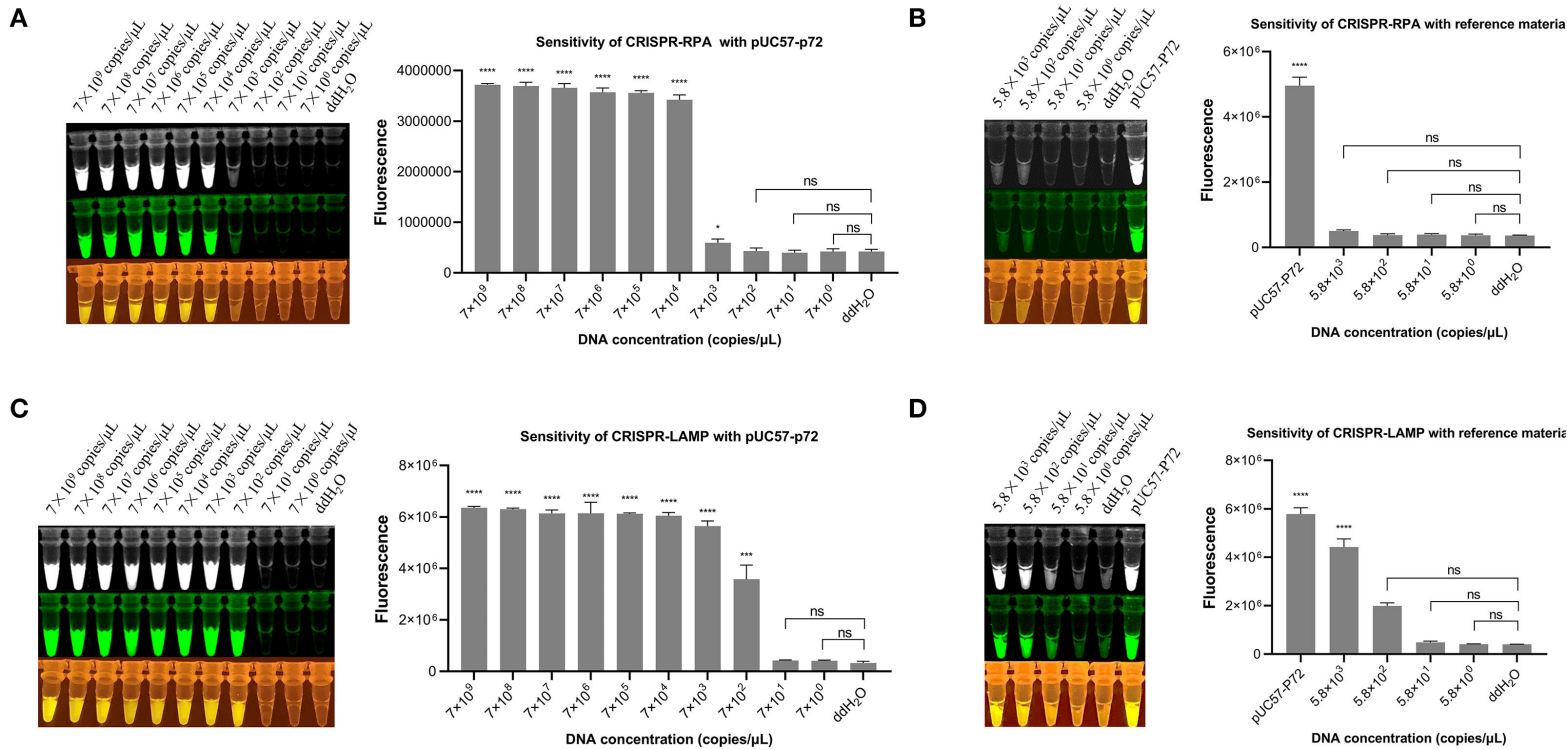
Sensitivity of one-pot visual detection system. **(A)** The sensitivity of CRISPR-RPA one-pot detection with 10-fold serial diluted pUC57-p72 DNA. **(B)** The sensitivity of CRISPR-RPA one-pot detection with 10-fold serial diluted ASFV *B646L* gene plasmid reference material. **(C)** The sensitivity of CRISPR-LAMP one-pot detection with 10-fold serial diluted pUC57-p72 DNA. **(D)** The sensitivity of CRISPR-LAMP one-pot detection with 10-fold serial diluted ASFV *B646L* gene plasmid reference material. Analysis of the value for the fluorescence image using the ImageJ software. Each measuring was run with three replicates (*n* = 3). *P < 0.05, **P < 0.01, ***P < 0.001, ****P < 0.0001, ns, not significant. Error bars represent the means ± s.d. from replicates. The unpaired two-tailed *t*-test was used to analyze the statistical significance.

On combining the RPA and CRISPR cleavage reactions, the sensitivity of the RPA-CRISPR reaction was determined with a 10-fold serial diluted template at a concentration of 7 × 10^9^, 7 × 10^8^, 7 × 10^7^,7 × 10^6^, 7 × 10^5^, 7 × 10^4^, 7 × 10^3^, 7 × 10^2^, 7 × 10^1^, and 7 × 10^0^ copies/μl of the pUC57-p72 DNA. The results showed that the developed RPA-CRISPR one-pot visual detection system can detect as low as 7 × 10^4^ copies/μl of the dsDNA template (Figure 4A). The ASFV *B646L* gene plasmid reference material was diluted with a 10-fold serial at a concentration of 5.8 × 10^3^, 5.8 × 10^2^, 5.8 × 10^1^, and 5.8 × 10^0^ copies/μl to refine and accurate the limitation. The results showed that no significant fluorescence signal can be detected by the naked eye, meaning that the limit of the one-pot visual detection based on the RPA-CRISPR is 7 × 10^4^ copies/μl within 40 min (Figure 4B). Besides, the sensitivity of “one-step” in one-pot detection is 7 × 10^8^ copies/μl which is not an appropriate limit for virus detection (Supplementary Figure 1).

The limits of the LAMP-CRISPR detection system were determined using a 10-fold serial diluted pUC57-p72 DNA template, ranging from 7 × 10^9^ to 7 × 10^0^ copies/μl. The results showed that the developed LAMP-CRISPR one-pot visual detection system can detect as low as 7 × 10^2^ copies/μl of the dsDNA template (Figure 4C). Using diluted ASFV *B646L* gene plasmid reference material as a template, the limit of one-pot visual detection system based on the LAMP-CRISPR was 5.8 × 10^2^ copies/μl within 60 min, as shown in (Figure 4D).

### Evaluating Consistency Between the One-Pot Visual Detection and qPCR

We further compared one-pot visual detection with the quantitative PCR (qPCR), the most commonly used detection method, as the gold standard. The same target on *p72* gene was used for the RPA and LAMP amplification to determine the analytical sensitivity of the qPCR. Serial dilutions were prepared from 10^9^ to 10^0^ copies/μl. The result showed that the limit of qPCR detection was 7 × 10^2^ copies/μl (Figure 3D). Compared to qPCR detection, the limit of one-pot detection was similar and the CRISPR-LAMP could attain a sensitivity of 5.8 × 10^2^ copies/μl. Moreover, both the CRISP-RPA and CRISPR-LAMP detection systems spent lesser detection time than the qPCR.

## Discussion

Since 2018, the rapid outbreak of ASF in China and in a number of other countries has resulted in tremendous economic losses (51). At present, molecular diagnostic techniques for detecting ASFV mainly rely on two OIE-recommended conventional and real-time qPCR technique methods. Although these techniques have been widely validated and are useful tools for detecting this disease, they remain inconvenient because of expensive instruments and professional operation systems. The CRISPR/Cas systems are revolutionary tools allowing for precise genome engineering, transcription regulation, and many other applications (52, 53). The Cas12a recognizes specific dsDNA sequences and then non-specifically cleaves the ssDNA, making it particularly suitable for detecting dsDNA viruses.

The cleavage site specificity of the Cas/crRNA complex is determined by the length of the crRNA and the sequence, number, location, and distribution of mismatch (54, 55). The 20nt or shorter nucleotide hybridizes with the dsDNA close to PAM region, which determines that CRISPR/Cas has high specificity (56). However, SNV in genome may cause off-target and false negatives when mutating on the target or the PAM (54, 57). The introduction of PAM through pre-amplification and multi-crRNA strategies may break the limitation caused by both PAM dependence and off-target, helping to improve the specificity of the detection system. The Cas12a enzyme itself has weak collateral cleavage activity; thus, it can only achieve low detection sensitivity without pre-amplification (27, 58). Therefore, in combination with isothermal amplification, such as RPA, LAMP, strand displacement amplification (SDA) (59–61), rolling circle amplification (RCA) (62, 63), exponential amplification reaction (EXPAR) (64, 65), and recombinase-aided amplification (RAA) (48, 66–68), we found that Cas12a was able to detect pathogens with high sensitivity and precision. In this study, we found that the CRISPR-RPA system is not highly sensitive, but it is faster than CRISPR-LAMP, which means that CRISPR-RPA is possible for rapid qualitative detection on the grassroots level.

Aerosol pollution, improper operation, and complex detection environment may unavoidably cause false positives in point-of-care testing (POCT), and two separate steps in CRISPR-based detection may make it more serious. However, one-pot detection could avoid false positives and high background positives caused by cross contamination (69–72). This means that template amplification, Cas-mediated enzyme cleavage reaction, and signal output are completed in one tube without opening or closing the cover (71, 73, 74). Moreover, to report the presence of target DNA, ssDNA probe linking a fluorophore (FAM in this study) and a quencher was used. After the indiscriminate cleavage was triggered, the fluorophore on the ssDNA probe was released and detected by specific wavelength light, making it visible and suitable for POCT, thereby avoiding the problems described previously (74–76). The contradiction between sensitivity and specificity in the detection process is thus solved to a certain extent.

Depending on expensive thermocyclers and skilled operator, PCR as the gold standard is difficult to popularize in POCT. Therefore, lower cost and personnel requirement detection methods could be more advantageous and required. Strip-based lateral flow assay (LFA) is an option with low cost (20, 77, 78), but opening tube while inserting the strip makes it less suitable. Therefore, the CRISPR-isothermal amplification method used in this study shows more advantages in POCT. A hand warmer and water bath, which gets rid of thermocyclers, can satisfy the meet for pre-amplification. Besides, the fixed wavelength illuminant makes the results more intuitive, clear and reduces the dependency on skilled operators, which greatly reduces the cost of POCT.

In conclusion, a one-pot visual detection system was established and used for the rapid, sensitive, specific, and low-cost detection of ASFV. This integration has great potential for POCT detection of ASFV and other DNA-based pathogens, which could be an effective way for the timely monitoring of ASFV to prevent its occurrence and spread at an early stage.

## Data Availability Statement

The original contributions presented in the study are included in the article/Supplementary Material, further inquiries can be directed to the corresponding authors.

## Author Contributions

KX and MZ contributed to the conceptualization and biosensor design, fabrication, and manuscript preparation. CQ performed the experiments, data curation, and drafted the manuscript. JL, WZ, JD, HZ, JZ, YK, LL, GS, ZL, HL, SJ, and KC performed formal analysis, validation support, and manuscript proofreading. XD and HM reviewed, edited, and supervised this work. All authors have read and agreed to the published version of the manuscript.

## Funding

This research was funded by the Agri-X Fund of Shanghai Jiao Tong University (Grant No. AF1500088/001/001).

## Conflict of Interest

The authors declare that the research was conducted in the absence of any commercial or financial relationships that could be construed as a potential conflict of interest.

## Publisher's Note

All claims expressed in this article are solely those of the authors and do not necessarily represent those of their affiliated organizations, or those of the publisher, the editors and the reviewers. Any product that may be evaluated in this article, or claim that may be made by its manufacturer, is not guaranteed or endorsed by the publisher.
