# Supplementary material for: One-Pot Visual Detection of African Swine Fever Virus Using CRISPR-Cas12a
Source: Front Vet Sci. 2022 Jul 18;9:962438. doi: 10.3389/fvets.2022.962438 (PMC9339671; doi:10.3389/fvets.2022.962438)
Supplement: Supplementary file 1 [file Data_Sheet_1.docx]

Supplementary Material

**Table S1**. primers for PCR and qPCR

| Name | Sequence (5’-3’) |
| --- | --- |
| PRV-F | CGGGCCGTGTTCTTTGT |
| PRV-R | GGTAGATGCAGGGCTCGTAC |
| PRRSV-F | CTAGGCCGCAAGTACATTCTG |
| PRRSV-R | TTCTGCCACCCAACACGA |
| PEDV-F | GGCTATTCTATGGATACTTTGGC |
| PEDV-R | ACCGCACTCGGATTACTCAC |
| PDCoV-F | CTGAACACCAGGCACATGTC |
| PDCoV-R | CTACTCATCCTCAGTTTCGT |
| qPCR primer-F | GGAACTAGTGGCCCTCTCCT |
| qPCR primer-R | CACGTTCGCTGCGTATCATT |

**Figure S1.** Optimization and sensitivity test of “one-step” in one-pot detection

**
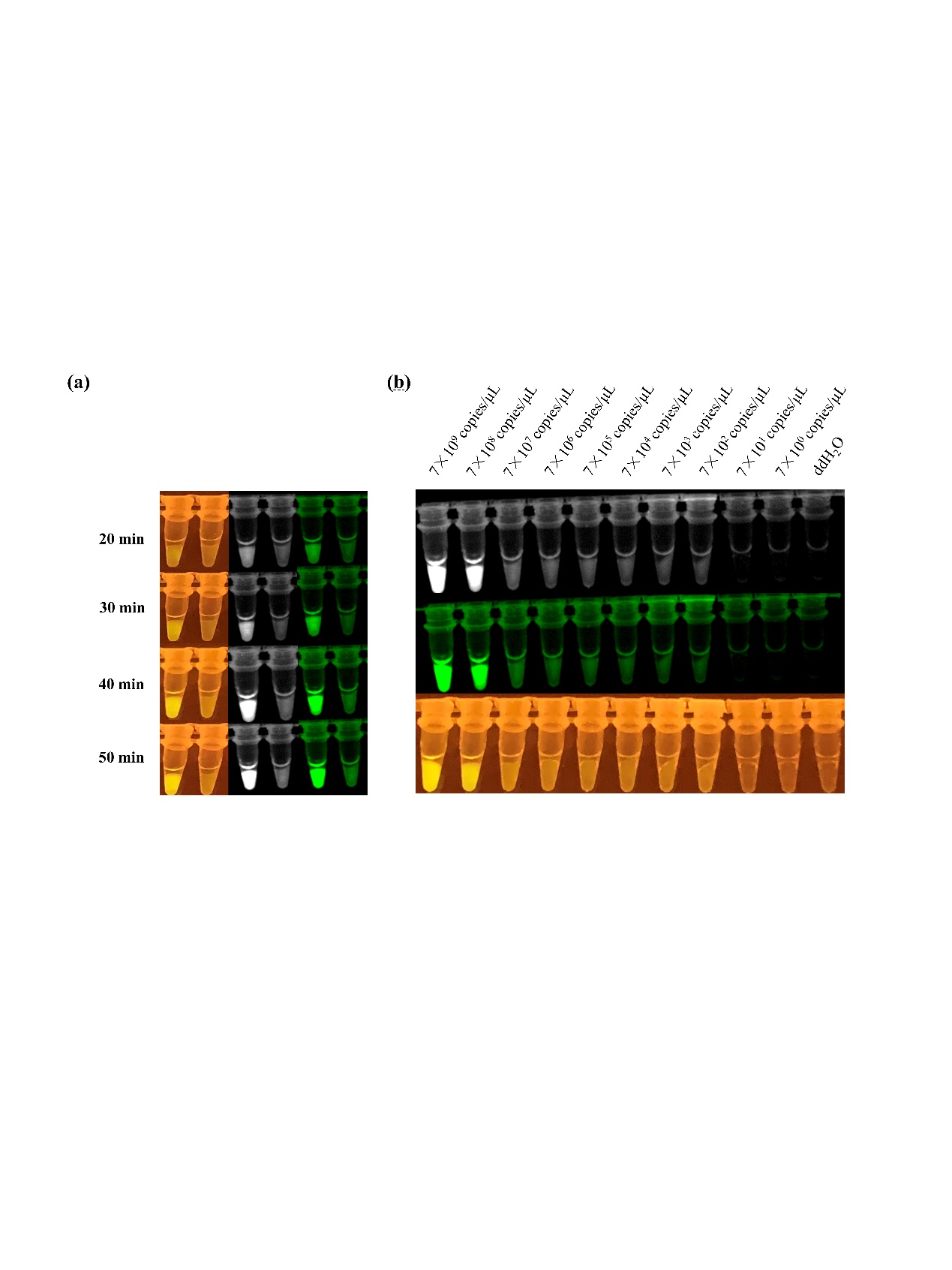
**
